# Current Advances in Immunoassays for the Detection of β_2_-Agonists

**DOI:** 10.3390/foods11060803

**Published:** 2022-03-11

**Authors:** Shuyu Ouyang, Shuting Yu, Yingying Le

**Affiliations:** 1 CAS Key Laboratory of Nutrition, Metabolism and Food Safety, Shanghai Institute of Nutrition and Health, University of Chinese Academy of Sciences, Chinese Academy of Sciences, Shanghai 200031, China; ouyangshuyu2018@sibs.ac.cn (S.O.); yushuting2019@sibs.ac.cn (S.Y.); 2 Key Laboratory of Food Safety Risk Assessment, Ministry of Health, Beijing 100021, China

**Keywords:** β_2_-agonist, sample extraction, immunoassay, radioimmunoassay, ELISA, chemiluminescent immunoassay, lateral flow immunoassay, immunosensor, food safety

## Abstract

β_2_-agonists are a group of synthetic phenylethanolamine compounds which are traditionally used for treating bronchospasm. These compounds can also increase skeletal muscle mass and decrease body fat. The illegal use of β_2_-agonists in food-producing animals results in residue of β_2_-agonists in edible tissues and causes adverse health effects in humans. Thus, the detection of β_2_-agonists at trace level in complex sample matrices is of great importance for monitoring the abuse of β_2_-agonists. Many methods have been developed to detect β_2_-agonists. Among them, a variety of antigen–antibody interaction-based techniques have been established to detect β_2_-agonists in various samples, including animal feed, urine, serum, milk, tissues and hair. In this review, we summarized current achievement in the extraction of β_2_-agonists from testing samples and detection of β_2_-agonists using immunological techniques. Future perspectives were briefly discussed.

## 1. Introduction

β_2_-agonists are a group of synthetic compounds with phenylethylamine structures, which are commonly used to treat asthma and chronic obstructive pulmonary disease [1]. They relax the smooth muscle of respiratory tract by combining to β_2_ adrenoreceptor. β_2_-agonists are also powerful anabolic agents which can promote protein synthesis, increase muscle mass and decrease fat tissue [2]. They are illegally used in food-producing animals as the growth promoters and nutrient repartitioning agents to escalate lean muscle gain, increase growth rate and feed efficiency [3,4,5,6]. Most countries around the world ban the use of all β_2_-agonistsin livestock feed and have established strict surveillance programs to ensure the food and feed safety. However, the illegal use of β_2_-agonists in livestock still happens, and the poisoning incidents caused by consumption of edible tissues from livestock bred with β_2_-agonistsare reported from time to time in countries around the world [7,8,9,10,11,12].

In order to monitor the illegal use of β_2_-agonists, various techniques have been developed to detect β_2_-agonists in animal samples (tissues, milk, urine, hair, etc.), including chromatography, spectrometry and related techniques [13,14], immunoassays [13,14], biosensors [14,15] and β_2_ adrenoreceptor-based assays [16,17]. Immunoassays are widely used in the purification and measurement of β_2_-agonists. The antibodies against β_2_-agonist can be prepared with β_2_-agonist hapten composed of β_2_-agonist and a carrier protein, such as serum albumin from bovine, human and rabbit, ovalbumin, keyhole limpet hemocyanin and bovine thyroglobulin. In this review, we summarize antigen–antibody interaction-based methods to purify and determine β_2_-agonists, including extraction of β_2_-agonists from samples through immunoaffinity chromatography, immunofiltration and immunomagnetic separation, and detection of β_2_-agonists by radioimmunoassay, enzyme linked immunosorbent assay (ELISA), chemiluminescence immunoassay, lateral flow immunoassay, immunosensors and other types of immunoassays.

## 2. β_2_-Agonist Antibody-Based Sample Extraction/Cleanup

Extraction and cleanup are important steps for the detection of β_2_-agonists in complex biological samples. Various techniques have been developed to extract and cleanup β-agonists, such as liquid–liquid extraction, solid phase extraction, matrix solid-phase dispersion, dialysis, supercritical fluid extraction [13,14,18]; and antibody-based immunoaffinity chromatography [18,19,20,21,22], immunofiltration [23,24,25] and immunomagnetic separation [26,27,28,29]. Antibody-based techniques provide better cleanup of the samples and higher selectivity than aforementioned other techniques and were summarized herein.

### 2.1. Immunoaffinity Chromatography

Immunoaffinity chromatography (IAC) is a technique that relies on antigen–antibody interactions to extract the analyte(s) of interest. Analyte from the sample is retained on the column containing immobilized antibody and eluted using minimal amounts of organic solvent. IAC has been accepted as an extractionpreconcentration procedure for detecting β_2_-agonists in biological samples owing to its high specificity and sample cleanup efficiency. IAC has been applied to extract clenbuterol, salbutamol, ractopamine and its metabolites from urine and tissue samples, respectively. Then, the target compound was detected using different techniques, including high-performance liquid chromatography (HPLC), electrochemical detection and capillary liquid chromatography-tandem mass spectrometry [19,20,21]. Lin et al. [22] developed a method to simultaneously detect clenbuterol, salbutamol, ractopamine and terbutaline in beef by IAC extraction and ultra-high-performance LC-MS/MS detection of these compounds. The immunoaffinity column was made by simultaneously covalent coupling of monoclonal antibodies against clenbuterol, salbutamol and ractopamine, respectively. As the antibodies are not specific for terbutaline, the limit of detection (LOD) of terbutaline is higher than that of the other three β_2_-agonists.

### 2.2. Immunofiltration

Immunofiltration has been applied for sample cleanup for detecting β_2_-agonists. The antibodies against β_2_-agonist are mixed with the samples and incubated in an ultra-filtration device. After centrifugation, the filter is washed with buffer, and the antibody bound β_2_-agonist is freed from the antibody by acetic acid. Immunofiltration was used to pretreat urine samples for detection of clenbuterol with a biosensor immunoassay [23] or ELISA [24]. Haasnoot et al. [25] reported that the anti-salbutamol polyclonal antibodies (pAb) recognized several β-agonists, and the combination of immunofiltration of β_2_-agonists with the ELISA could detect at least ten β_2_-agonists in urine with comparable LODs.

### 2.3. Immunomagnetic Separation

Immunomagnetic separation involves the coupling of biological macromolecules, such as antibodies and streptavidin, to superparamagnetic particles. When added to a heterogeneous target suspension, the magnetic particles bind to the desired target and form a complex which can be removed from the suspension by using a magnet. Immunomagnetic separation has been used as a sample pretreatment technology for purification and enrichment of β_2_-agonists from samples. Chen et al. [26,27] prepared immunomagnetic beads using monoclonal antibodies against clenbuterol and salbutamol, respectively, purified these compounds from animal urine samples and detected them by surface-enhanced Raman spectroscopy. Peng et al. [28] separated clenbuterol from swine urine samples using immunomagnetic particles which was prepared by immobilizing biotinylated clenbuterol monoclonal antibody (mAb) on the streptavidin magnetic nanoparticles through biotin–streptavidin interaction. Huang et al. [29] modified the fluorescent magnetic nanobeads with anti-clenbuterol mAb to specifically recognize and capture clenbuterol in swine urine. The fluorescent magnetic nanobeads not only served as a carrier for immunomagnetic separation of clenbuterol but also as a fluorescent probe for fluorescent lateral flow immunoassay of clenbuterol.

The advantages and disadvantages of the methods for extracting β_2_-agonists from samples using antibodies are summarized in Table 1.

## 3. Immunological Techniques for Detecting β_2_-Agonists

Many immunological methods based on an antigen–antibody interaction have been developed for qualitative/quantitative analysis of β_2_-agonists in biological samples, including radioimmunoassay, ELISA, chemiluminescence immunoassay, lateral flow immunoassay, immunosensors and other types of immunoassays.

### 3.1. Radioimmunoassay

The classical radioimmunoassay (RIA) is based on the principle of competitive binding. The unlabeled antigens and a fixed amount of radiolabeled antigens compete for a limited amount of antibody to form an antigen–antibody complex. The amount of labeled antigen–antibody complex formed is inversely related to the concentration of the unlabeled antigen. RIA has been applied to detect β_2_-agonists, such as salbutamol, fenoterol and clenbuterol in various samples, including plasma/serum, urine, feces and liver tissue [30,31,32,33,34]. If the pAb against one β-agonist has cross-reactivity with other β-agonists, the RIA can be applied as a qualitative method to detect the presence of one or more β-agonists [33,34], then a confirmatory analysis would be performed to determine the concentration of the analyte(s). The use of RIAs in detecting β_2_-agonists has been largely replaced by nonisotopic immunoassays because of concerns over the safe handling and disposal of radioactive reagents and waste.

### 3.2. ELISA

ELISA, also referred to as enzyme immunoassay, is a plated-based solid-phase enzyme immunoassay commonly used to identify the presence and concentration of antibodies or antigens in liquid samples. Detection of antigens is accomplished by binding enzyme–antibody conjugates to the antigen in the sample and assessing the enzyme activity via incubation with a substrate to produce a measurable end product. A number of enzymes are used in ELISA, including horseradish peroxidase (HRP), alkaline phosphatase (ALP) and β-galactosidase. ELISA can be classified into direct, indirect, sandwich and competitive ELISA. The competitive ELISA is very useful for determining the concentration of small-molecule antigens in complex sample mixtures. Direct and indirect competitive ELISAs have been developed to detect β_2_-agonist in various samples.

#### 3.2.1. Direct Competitive ELISA

Clenbuterol [35,36], salbutamol [37] and phenylethanolamine A [38] in various samples (animal urine, blood, hair and tissues) have been measured using direct competitive ELISA. The mAb or pAb against β_2_-agonist is coated onto the microtiter plate, the β-agonist in sample and β_2_-agonist labeled with HRP or ALP competes for binding with the mobilized antibody. Addition of the substrate of the enzyme yields a signal that is inversely proportional to β-agonist concentration within the sample. Huang et al. [39] developed a direct competitive ELISA using bacteria-Au-antibody/HRP composite as a probe to detect clenbuterol. The clenbuterol in the sample competed with clenbuterol immobilized on a plate for binding to the limited probe. The use of bacteria as a carrier and Au nanoparticles as cross-linking agents enriched HRP and reduced the antibody applied, which resulted in the amplification of signal in detection. The liner range and limit of detection (LOD) of clenbuterol were 0.02–1.0 ng/mL and 0.03 ng/mL, respectively.

#### 3.2.2. Indirect Competitive ELISA

Indirect competitive ELISAs have been developed to detect β_2_-agonists, including phenylethanolamine A [40], ractopamine [41], zilpaterol [42], clenbuterol [43,44] and salbutamol [45,46] in various types of samples. In these assays, the β_2_-agonist coated on the microtiter plate competes with β_2_-agonist in samples for binding to the primary anti-β_2_-agonist antibody. Probe labeled secondary antibody against the primary antibody was added successively. The signal produced by the secondary antibody is inversely proportional to β_2_-agonist concentration within the sample. Most of the above indirect competitive ELISAs used HRP to label the secondary antibody. Fang et al. [46] used Eu^3+^-labeled secondary antibody in the ELISA to detect salbutamol. Han et al. [41] developed a gold nanoparticle-based indirect competitive ELISA to detect ractopamine by using a secondary antibody labeled with catalase through biotin–streptavidin interaction. In the presence of ractopamine in the sample, gold(III) ions are oxidized by H_2_O_2_ to form red AuNPs. In the absence of ractopamine, the AuNPs in the solution are purple or blue due to aggregation. The LOD for ractopamine in urine was 0.35 ng/mL. In general, the primary antibody specifically against one β_2_-agonist was used in the development of the indirect competitive ELISA. Wang et al. [44,45] developed indirect competitive ELISAs using polyclonal R-(-)-salbutamol antibody which could recognize 31 β-agonists and analogues, and clenbuterol monoclonal antibody which could recognize 23 β-agonists and analogues, respectively. Holographic and three-dimensional quantitative structure–activity relationship models were developed for predicting the epitopes on *β*-agonist hapten affecting antibody specificity, which will contribute to the rational design and control of the immunoassay specificity of β_2_-agonist.

### 3.3. Chemiluminescence Immunoassay

Chemiluminescence describes the emission of light that occurs as a result of unique chemical reactions. In a chemiluminescence reaction, energy is released in the form of photons when electronically excited molecules, produced as a result of the reaction, relax to a stable ground state. Chemiluminescence immunoassay (CLIA) combines chemiluminescence technique with immunochemical reactions using luminescent chemical which could generate light emission. In CLIA, the luminescent chemical is used to label antigen, antibody or used as substrate of enzymes. HRP and ALP are the most widely used enzymes. Luminol and AMPPD are common chemiluminescent substrates used for HRP and AP, respectively.

CLIAs have been developed based on competitive immunoassay to detect salbutamol and brombuterol, respectively. HRP-labeled secondary antibodies were introduced into CLIAs [47,48]. The chemiluminescence intensity was linearly related to the concentration of salbutamol in the range of 0.5–100 ng/mL with a LOD of 0.15 ng/mL [47]. The LOD of brombuterol was 0.33 pg/mL [48]. Wang et al. [49] developed a competitive chemiluminescence immunosensor based on a microfluidic chip to detect ractopamine in swine urine samples using HRP-labeled secondary antibody. The immunosensor could provide a liner range of 0.5–40 ng/mL with a LOD of 0.97 ng/mL for ractopamine detection.

### 3.4. Lateral Flow Immunoassay

Lateral flow immunoassay (LFIA), also known as immunochromatographic assay, is a combination of chromatography and immunoassay using a nitrocellulose membrane as a support to detect labeled antigen–antibody complexes in liquid samples. The signal produced on the membrane can be evaluated by the naked eye or with the aid of portable devices. Direct assay (sandwich assay) and competitive assay are two standard formats of LFIA, which are used to detect large and small molecules, respectively. Competitive tests have been used to detect β_2_-agonists. A variety of tracers that cause color or optical changes following antigen–antibody interactions have been used in LFIA to detect β_2_- agonists, such as nanoparticles, fluorescent nanomaterials and upconverted phosphorus nanoparticles. Compared with traditional detection methods, LFIA possesses significant advantages, including simple fabrication, low cost, fast and simple analytical procedure.

#### 3.4.1. Colorimetric LFIA

Nanoparticles with specific structures can produce color by assemblies and aggregations. Various nanoparticles with specific properties have been used as tracers for LFIA to detect β_2_-agonists in samples.

##### Gold Nanoparticles as Tracers

Colloidal gold or gold nanoparticle is the most widely used label in LFIA for detecting β_2_-agonists in samples. It has an intense color and no development process is needed for visualization. Colloidal gold has been used by multiple laboratories to label an antibody against β_2_-agonist and develop LFIA for detecting target β_2_-agonist in biological samples, such as clenbuterol in urine and pork muscle [50,51], salbutamol in urine, meat and milk [52,53], phenylethanolamine A in urine and pork [40,54] and ractopamine in animal urine, meat, liver and feed [50,55,56,57,58]. Huang et al. [59] used bacteria as a carrier of gold nanoparticles to construct bacteria-Au-antibody probe in LFIA to detect clenbuterol. Much fewer antibodies are needed in the probe to produce a clearly visible color. The visual detection limit (VDL) for clenbuterol is 0.1 ng/mL in urine, 0.5 ng/mL in milk and 0.2 ng/g in swine feed. Wang et al. [60] developed silver–gold nanoparticles (Au-Ag NPs) probe-based LFIA to detect clenbuterol. The quantitative detection limit of optimized hollow Ag-Au NPs labeled lateral-flow immunochromatographic test strip approaches to 2 ppb. Compared with Au NPs and Ag NPs-labeled test strips, Ag-Au hollow NPs-labeled test strips exhibited much higher sensitivity for qualitatively detecting clenbuterol. Chen et al. [61] established an integrated LFIA to detect clenbuterol using anti-clenbuterol antibody labeled with colloidal gold and fluorescent nanobeads, respectively. The integrated test strip could qualitatively and quantitatively detect clenbuterol with VDL of 0.5 ng/mL and LOD of 0.04 ng/mL.

##### Other Nanoparticles as Tracers

Colored silica nanoparticles (SiNPs) have been used as a visible label for LFIA to detect β_2_-agonists. Zhu et al. [62] developed LFIA to detect clenbuterol using purple SiNPs labeled anti-clenbuterol antibody. The VDL for clenbuterol were 6 ng/mL and 5 ng/mL in urine and pork, respectively. Yu et al. [63] developed colored SiNP-based LFIA to detect clenbuterol and ractopamine simultaneously by using anti-clenbuterol antibody and anti-ractopamine antibody labeled with red and blue SiNPs, respectively. The visible limit of detections for clenbuterol and ractopamine were 3 ng/mL and 2 ng/mL, respectively. Wang et al. [64,65] used selenium nanoparticles to label antibodies against clenbuterol, ractopamine and salbutamol, respectively, and developed LFIAs to detect these compounds in swine urine. Zhao et al. [66] developed LFIA for clenbuterol detection using Prussian blue nanoparticles-labeled antibody against clenbuterol. The VLDs for clenbuterol were 3.0 ng/mL in pork, 5 ng/g in both swine kidney and bacon samples. Liu et al. [67] used ultramarine blue nanoparticles as visible labels in LFIAs to detect ractopamine. The visual limit of detection for ractopamine is 2.0 ng/mL, and 1.0 ng/mL in feed and pork samples, respectively.

#### 3.4.2. Luminescent LFIA

Luminescent materials, such as fluorescent nanomaterials and up-converting phosphorus nanoparticles, have been used as reporter to develop sensitive LFIA for detecting β_2_-agonists.

Various kinds of fluorescent nanoparticles have been used as probes to develop LFIA for detecting β_2_-agonists. Song et al. [68] used fluorescent nanosilica conjugated mAb against clenbuterol as a signal probe to build a LFIA for detecting clenbuterol. The VLD for qualitative detection was 0.1ng/mL and the LOD for quantitative detection was 0.037 ng/mL, which was much better than the colloidal gold-based strip. Huang et al. [29] developed an LFIA to detect clenbuterol using fluorescent magnetic nanobeads (FMNBs) to label anti-clenbuterol mAb. The FMNBs-Ab probe worked as a carrier for immunomagnetic separation of clenbuterol from sample and as a fluorescent label for the LFIA. The LOD of clenbuterol in swine urine is 0.22 ng/mL. Wang et al. [69] developed fluorescent beads-based LFIA to simultaneously detect 3 β_2_-agonists. Fluorescent beads were conjugated with mAb specific for clenbuterol, ractopamine and salbuterol, respectively, and put on the conjugated pad. There were three test lines in the analytical pad, which contained immobilized clenbuterol, ractopamine and salbuterol, respectively. The LODs for clenbuterol, ractopamine and salbuterol were 0.10, 0.10 and 0.09 ng/mL, respectively, which were better than that of the colloidal gold-based strip. The same lab developed an LFIA to detect clenbuterol and its structural analogues in pork, using fluorescent nanoparticle-conjugated anti-clenbuterol antibody which had cross-reactivity with mabuterol, brombuterol, cimaterol, cimbuterol, bromchlorbuterol and banbuterol [70]. The established LFIA could screen these seven β_2_-agonists in a single run. The LODs for these compounds in pork were <50 pg/g. Hu et al. [71] compared time-resolved fluorescent nanobeads, fluorescent submicrospheres, quantum dots and colloidal gold-based LFIA (TRFN-LFIA, FM-LFIA, QD-LFIA, and CG-LFIA) for detection of ractopamine in swine urine. TRFN-LFIA showed the highest sensitivity (LOD 7.2 pg/mL) and a wide linear range of detection (5–2500 pg/mL). In addition, TRFN-LFIA exhibited the shortest detection time compared with the other LFIAs. Shi et al. [72] developed a fluorescence quenching-based LFIA for detecting ractopamine. The antibody against ractopamine was labeled with gold nanoparticles (AuNPs) and loaded on the conjugate pad. The test line was coated with Rac and fluorescent polymer dots (FPDs). The control line was coated with FPD. Adding a negative sample resulted in the binding of Ab-AuNPs to ractopamine immobilized on T-Line, which in turn caused fluorescence resonance energy transfer between the FPDs and AuNPs and quenches fluorescence. Adding a positive sample caused a reaction between the ractopamine analyte and Ab-AuNPs and visible fluorescence at the test line. The intensity of fluorescence at the test line positively reflected the amount of ractopamine in the sample. The LOD was 0.16 ng/mL.

Wang et al. [73] developed an LFIA based on upconversion phosphor (UCP) for detection of clenbuterol. The mAb against clenbuterol was labeled with UCP beads which can emit higher energy light under excitation of lower energy light. After reaction, luminescence from clenbuterol-mAb-UCPs can be detected by a scanning photometer or naked eyes. The visual limit of detection for clenbuterol was 0.1 ng/mL. An LFIA based on a time-resolved chemiluminescence was established to detect ractopamine and clenbuterol simultaneously [74]. Antibodies against ractopamine and clenbuterol were conjugated with HRP and ALP, respectively. As the reaction kinetics of HRP and ALP chemiluminescent reaction systems were different, ractopamine and clenbuterol could be sequentially detected in different time windows. The LODs for ractopamine and clenbuterol were 0.17 ng/mL and 0.067 ng/mL, respectively.

#### 3.4.3. Other Types of LFIA

Zhang et al. [75] developed a competitive LFIA to detect clenbuterol in milk, swine liver and tenderloin using Coomassie Brilliant Blue (CBB)-stained anti-clenbuterol antibody. The CBB-antibody was used as both a recognition reagent and a chromogenic probe, enabling the simple but sensitive detection of clenbuterol with LOD of 2 ng/mL.

Surface-enhanced Raman scattering (SERS)-based LFIA has been developed for the detection of β_2_-agonists, such as phenylethanolamine A [76], clenbuterol [77] and brombuterol [78]. The principle of this method is similar to the LFIA based on colloidal gold particles, but the anti-β_2_-agonist antibody was labeled with Au@Ag core–shell nanoparticles sandwiched with a Raman reporter (4-mercaptobenzoic acid, MBA) [76,77], or with flower-like gold–silver core–shell bimetallic nanoparticles carrying the MBA [78]. The LOD values for detection of phenylethanolamine A, clenbuterol and brombuterol were 0.32, 0.24 and 0.5 pg/mL, respectively [76,77,78].

### 3.5. Immunosensors

Immunosensors use antibodies as the recognition element and a transducer that converts the antigen–antibody binding event to a measurable physical signal. Surface plasmon resonance, surface-enhanced Raman scattering, electrochemiluminescence and electrochemical immunosensor are types of immunosensors developed for detecting β_2_-agonists.

#### 3.5.1. Surface Plasmon Resonance Sensor

Surface plasmon resonance (SPR) is a phenomenon occurring at metal surfaces when plane-polarized light hits a metal film under total internal reflection conditions. SPR signal is directly dependent on the refractive index of the medium on the metal film. The binding of biomolecules on the metal surface results in changes in the refractive index. As SPR allows real-time, label-free detection of biomolecular interactions, it has been used as a powerful tool to study interactions between biomolecules, such as the interaction of antibody and antigen, ligand and receptor, enzyme and substrate [79].

A number of investigators developed competitive immunoassays to detect β_2_-agonists using SPR sensors. A known concentration of β_2_-agonist antibody was mixed with the sample and injected over the surface of the sensor chip. The β_2_-agonist in the sample bound to the antibody and inhibited it from binding to the β_2_-agonist mobilized on the sensor chip surface and consequently induced an increase in the refractive index at the SPR sensor surface. SPR sensor has been built to detect salbutamol in swine urine with LOD of 5 ng/mL [80]. Suherman et al. [81] fabricated SPR sensors with covalently immobilized β-albuterolvistion on Au chips for detection of ractopamine and salbutamol, respectively. The LODs for ractopamine and salbutamol were 10 pg/mL and 5 pg/mL, respectively. The sensor surface could be regenerated and reused more than 100 times. Wang et al. [82] developed a SPR to detect ractopamine in swine urine through indirect competitive assay, with an LOD of 0.09 ng/mL. Kabiraz et al. [83,84] built SPRs sensors to detect clenbuterol using Au nanoparticles to label the primary antibody against clenbuterol or the secondary antibody. The LODs were 0.05 pg/mL and 100 fg/mL, respectively, which were lower than that of unlabeled Ab. 

#### 3.5.2. Surface-Enhanced Raman Scattering-Based Immunosensor

Surface-enhanced Raman scattering (SERS) is a powerful spectroscopy technology that can provide a nondestructive and ultra-sensitive characterization down to single molecular level. Competitive SERS immunoassay has been applied to detect β_2_-agonists, based on the competition between free β_2_-agonists in samples and β_2_-agonists immobilized on the solid substrate for binding to antibodies on SERS nanoprobes. The intensity of SERS signal is conversely correlated with the concentration of β_2_-agonist in sample. The SERS nanoprobe is prepared by labeling Au nanoparticles (AuNPs) with Raman reporter and anti-β_2_-agonist antibody. 4,40-dipyridyl and 2,20-dipyridyland 4-mercaptobenzoic acid (MBA) have been used as Raman reporters to prepare SERS nanoprobes for detecting clenbuterol and ractopamine, respectively [85,86]. Gu et al. [87] developed a SERS-based competitive immunoassay for detecting salbutamol and brombuterol in swine meat, liver and human urine. The SERS probe was prepared by immobilizing antibody against salbutamol (or brombuterol) and SERS reporter nile blue (or 3,3′,5,5′ -tetramethylbenzidine)-labeled DNA concatemers on gold nanoparticles. The introduction of long DNA concatemers in SERS probe and stain of the immune complex with Ag nanoparticles after competitive immunoreaction greatly enhanced Raman signals. The LODs for salbutamol and brombuterol were 2.0 pg/mL and 1.0 pg/mL, respectively. Wei et al. [88] developed a SERS-based liquid magnetic competitive immunoassay to detect clenbuterol. The SERS probe was prepared by labeling AuNPs with Raman reporter MBA and clenbuterol antibody. Fe_3_O_4_@Au nanoparticles were labeled with clenbuterol. After competitive reaction, the immunocomplex of SERS probe and Fe_3_O_4_@Au-clenbuterol were enriched by an external magnetic field and the SERS signal was collected. The LOD for clenbuterol was 0.22 fg/mL. Yao et al. [89] developed a SERS/resonance Rayleigh scattering (RRS) dual-spectroscopic immunosensor to detect clenbuterol based on nitrogen/silver-codoped carbon dots (CDN/Ag) catalytic amplification. CDN/Ag can strongly catalyze trisodium citrate-HAuC_4_ reaction to generate red nanogold that exhibits a strong RRS signal. After adding Victoria blue B (VBB), the system also exhibits a strong Raman signal. Electrostatic coupling between CDN/Ag and Ab inhibits the catalytic activity of CDN/Ag and reduces the Raman signal. In the presence of clenbuterol, the binding of Ab with clenbuterol reduces the interaction of Ab with CDN/Ag and restore the catalytic activity of CDN/Ag, which enhances the Raman signal through increasing gold nanoparticles production. The SERS/RRS immunosensor detected clenbuterol with an LOD of 0.68 pg/mL.

#### 3.5.3. Electrochemiluminescence Immunosensor

Electrochemiluminescence (ECL) is the process whereby species generated at the electrode undergo a high-energy electron transfer reaction to form excited states that emit light. ECL immunosensor combines immunoreaction and ECL, which can be used to quantitatively measure an antigen or antibody based on the change in ECL signal before and after immunoreaction. The ECL luminophore is one of the most significant components during the light generation processes. Various ECL luminophores have been reported, including organic, metal complexes, nanomaterials, metal oxides and near-infrared ECL luminophores [90]. Nanomaterials, such as quantum dots (QDs), have been adopted as final emission species either after direct oxidation reactions or after chemiluminescence resonance energy transfer.

Cadmium selenide (CdSe) QDs are one of the most popular ECL emitters due to their intrinsic properties, such as unique luminescent properties and relatively low cost. CdSe QDs have been used in ECL immunosensor to detect β_2_-agonists. CdSe QDs immobilized on an electrode surface could react with H_2_O_2_ to produce a high ECL emission. In the presence of HRP substrate, the consumption of H_2_O_2_ in the HRP-catalyzed oxidation process leads to quenching of ECL emission. HRP is conjugated to anti-β_2_-agonist Ab or the secondary Ab. In the competitive ECL immunoassay, β_2_-agonist in the sample competes with β_2_-agonist immobilized on the electrode for the primary Ab and reduces the amount of HRP-containing immunocomplex on the electrode, which alleviates the quenching of the ECL emission by reducing the consumption of H_2_O_2_ by HRP. Thus, the ECL intensity was proportional to the amount of β_2_-agonist in sample. ECL immunosensors based on CdSe QDs and HRP-labeled secondary Ab have been constructed to detect salbutamol in pork and liver, with an LOD of 8.4 pg/mL [91]. ECL immunosensors based on CdSe QDs and anti-salbutamol or anti-ractopamine Ab-conjugated AuNPs-HRP have been constructed to detect these compounds in pork, liver and feed. AuNPs provided a matrix to anchor a large amount of HRP which greatly enhanced the electrochemical quenching. LODs for salbutamol and ractopamine were 17 pg/mL and 1.7 pg/mL, respectively [92,93].

To construct ECL immunosensors to detect β_2_-agonist, many researchers used QDs as probe to label the anti-β_2_-agonist antibody and used AuNPs as substrates and electron transport accelerators. By competitive immunoassay, the β_2_-agonist in the sample competes with the β_2_-agonist immobilized on the electrode for binding to QDs labeled β_2_-agonist Ab, which results in the reduction in the β_2_-agonist-Ab-QDs complex on electrode and ECL signal. Dong et al. [94] used CdSe@SiO_2_ nanoparticles to label antibody against salbutamol to construct an ECL immunosensor to detect salbutamol in pork. The detection range was 0.001–1000 pg/mL with an LOD of 0.17 pg/mL. To amplify the ECL signal, various chemicals which can carry more QDs have been used to prepare the antibody probe for detecting β_2_-agonists. Dong at al. [95] used polyamidoamine dendrimers (PAMAM) and silver-cysteine hybrid nanoribbon (SNR) to prepare CdSe QDS-PAMAM-SNR-Ab for detecting brombuterol in pork and feed. Zhu et al. used CdTeQDs-PAMAM-GO-labeled antibody composite to detect brombuterol [96] and used CdSe QDs/PDDA-GN/AuNPs-labeled antibody composite to detect ractopamine in pork [97]. The amplification of the ECL signal increased the sensitivity of these ECL immunosensors and decreased the LOD for β_2_-agonist to lower pg/mL level.

Li et al. [98] developed an ECL resonance energy transfer (RET) immunosensor to detect brombuterol residues in pork and swine feed based on competitive immunoassay. The electrode was modified with graphitic-phase carbon nitride (g-C_3_N_4_) which worked as the ECL emitter. The antibody against brombuterol was labeled with Au-Ag alloy nanoparticles which were energy acceptors. When there is no brombuterol in the sample, the Au-Ag antibody binds with brombuterol immobilized on g-C_3_N_4_-coated electrode. Resonance energy transfer occurs and the ECL emission was quenched. On the contrary, brombuterol in the sample competed with brombuterol immobilized on g-C_3_N_4_ to bind Au-Ag antibody, g-C_3_N_4_ releases a strong ECL signal. The ECL-RET immunosensor for brombuterol detection exhibited high sensitivity with an LOD of 0.31 pg/mL.

#### 3.5.4. Electrochemical Immunosensor

Electrochemical immunosensors combine highly sensitive electrochemical sensing technology with highly specific immunological techniques. In electrochemical immunosensor, the antibody and antigen interaction results in the change in electrical signal, such as current, voltage, resistivity and impedance, that correlates with the concentration of analyte.

##### Label-Free Electrochemical Immunosensor

Highly sensitive label-free electrochemical immunosensors have been constructed by immobilizing anti-β_2_-agonist antibody on various types of electrodes which carry a gold nanostructure and other modifiers for detecting β_2_-agonist, such as salbutamol [99], ractopamine [100] and clenbuterol [101].The LOD was at pg/mL or fg/mL level. Cui et al. [102] constructed a sandwich-type electrochemical immunosensor for detecting salbutamol. The primary salbutamol antibody was immobilized onto sodium dodecylbenzene sulfonate-functionalized graphene sheets (SDBS-GS) and coated on the electrode. The secondary salbutamol antibody was conjugated to Pd@SBA-15/BMIM·Br nanoparticles. The electrochemical immunosensor exhibited a wide working range from 0.02 to 15.0 ng/mL with an LOD of 7 pg/mL. Wang et al. [103] constructed a multiplexed electrochemical biosensor to simultaneously detect ractopamine (RAC), salbutamol (SAL) and clenbuterol (CLB) through a competitive immunoassay. Artificial antigens of RAC, SAL and CLB were, respectively, immobilized onto three working electrodes by binding to reduced graphene oxide. The antibody against RAC, SAL or CLB was conjugated with silver-palladium alloy nanoparticles (AgPd NPs). β_2_-agonists in the sample competed with RAC, SAL and CLB on the electrodes for binding antibodies on AgPd NPs. This immunosensor can simultaneously detect RAC, SAL and CLB ranging from 0.01 to100 ng/mL with LOD of 1.52, 1.44 and 1.38 pg/mL, respectively. Gu et al. [104] developed three electrochemical immunosensors. Each can detect six β2-agonists through a competitive immunoassay. β_2_-agonists in the sample competed with ractopamine (RAC), clenbuterol (CL) or salbutamol (SAL) immobilized on graphene-coated glassy carbon electrode (GCE/GNP) to bind with pAb against BSA-RAC-CL-SAL antigen. As the antibody had cross-reactivity with terbutaline, mabuterol and tulobuterol, each immunosensor could detect six β_2_-agonists. All three immunosensors could detect CL with LODs of 0.1 ng/mL. The GCE/GNP/RAC immunosensor detected the other five β_2_-agonists with LOD of 0.1 ng/mL, which was lower than those of the other two immunosensors.

##### Enzyme-Labeled Electrochemical Immunosensors

Enzymatic-labeled electrochemical immunosensors have been constructed to detect β_2_-agonist based on competitive immunoassay, using electrode coated with β_2_-agonist or primary β_2_-agonist antibody/secondary antibody, and enzyme-labeled β_2_-agonist antibody or β_2_-agonist. The most commonly used enzymes are HRP and ALP. To enhance the electroactivity, the electrodes are usually modified with various materials, such as gold nanoparticles, polyaniline/poly(acrylic acid), colloidal Prussian blue and multiwall carbon nanotubes. Various nanomaterials are used as carriers of enzyme-conjugated β_2_-agonist/antibody to amplify the signal for β_2_-agonist detection.

Electrochemical immunosensors were developed to detect salbutamol by immobilizing salbutamol on the electrode modified with gold nanoparticles or other materials, and conjugating the HRP antibody to graphene, Au hybrid graphene nanocomposite or multiwall carbon nanotubes [105,106,107]. The LODs were between 0.03 ng/mL and 0.06 ng/mL, respectively. Electrochemical immunosensors have been fabricated to detect clenbuterol by immobilizing a primary or secondary antibody against clenbuterol on the electrode with various modifications, and labeling clenbuterol with HRP, ALP or glucose oxidase [108,109,110,111]. The LOD of clenbuterol is between 0.008 and 0.25 ng/mL.

### 3.6. Other Types of Immunoassays

In addition to the aforementioned immunological techniques, other methods based on the antigen–antibody interaction were also established for detecting β_2_-agonists, such as immune-PCR, gel-based immunoassay, fluorescence polarization immunoassay and integrated microfluidic immunoassay.

Immuno-PCR is a technique to detect antigens and antibodies through a combination of an immunoassay and a PCR. In 1992, Sano et al. [112] developed an immuno-PCR technique based on the direct ELISA, in which the detection enzyme in ELISA was replaced with a biotinylated reporter DNA bound to the antigen–antibody complex through a streptavidin-protein A fusion protein. It combined the antigen detection with ultrasensitive PCR signal amplification, which greatly increases the sensitivity of detection. In recent years, immuno-PCR techniques have been developed by combining versatile DNA-antibody linking methods with real-time PCR (qPCR) to detect β_2_-agonists. Lei et al. [113] established a direct competitive immuno-PCR assay to detect salbutamol in human urine. Each PCR tube was coated with salbutamol antibody, to which salbutamol labeled with double-stranded DNA and free salbutamol in the sample are competitively bound. The bound DNA in tubes was amplified and quantified by qPCR. Under the optimized conditions, the assay showed a linear range over seven orders of magnitude, whereas the LOD of salbutamol in the human urine sample was 28 fg/mL. Zhao et al. [114] developed a complete antigen-bridged DNA strand displacement amplification immuno-PCR assay for detecting salbutamol. Salbutamol antibodies were immobilized on the immunomagnetic beads. Salbutamol-BSA was linked to DNA probe 1 or 2 which each contained a split DNA barcode and a recognition site for cleavage endonuclease and subsequent quantification by qPCR. Upon binding with salbutamol antibodies on the immunomagnetic beads, the salbutamol probe 1 and probe 2 were closed and form a full-length DNA amplicon after the addition of a complementary bridging DNA and ligase. The full-length DNA amplicons were separated by magnetic separation and amplified by strand displacement amplification (SDA) and qPCR. To detect salbutamol, the salbutamol in the sample competes with salbutamol probes for binding salbutamol antibody, resulting in the reduction in full-length DNA amplicons necessary to SDA and qPCR. The developed immuno-PCR assay specifically detected salbutamol with LOD of 0.65 fg/mL. It was successfully applied to the detection of salbutamol in water and urine samples.

Li et al. [115] developed a gel-based immunoassay for detecting salbutamol and ractopamine residues in pork. The anti-salbutamol antibody and anti-ractopamine antibody were immobilized onto the Sepharose 4B gel, respectively. The anti-salbutamol gel and anti-ractopamine gel were loaded into 1 mL Bond Elut cartridges as the test layers, separated by a layer of air. The target analytes in the samples would compete with HRP-analyte conjugates to combine with the antibody in the test layer. After adding the chromogenic substrate, the negative samples presented a blue color on the respective test layers, while the positive samples presented no or negligible color. The color of the test layers was visually evaluated or photographed for color density-based quantitative analysis. The LODs were 0.5 μg/kg for salbutamol and ractopamine by visual detection. The quantitative LODs of salbutamol and ractopamine in spiked pork samples were 0.051 μg/kg and 0.02 μg/kg, respectively.

Fluorescence polarization immunoassay (FPIA) is based on measuring the polarization of light caused by changes in molecular size as a result of antigen–antibody reactions. FPIA use a fluorescein-labeled antigen to compete with the unlabeled antigen for an antibody. If the fluorescein-labeled antigen combines with the antibody, emitted light remains polarized when the incoming light is polarized. As the concentrations of antigen in the sample increase, free fluorescein-labeled antigen molecules also increase in number. Since the unbound fluorescein-labeled antigen molecule rotates freely, the polarized light emitted is reduced. FPIA has been widely applied to small molecule analysis. Zvereva et al. [116] detected ractopamine through FPIA, using ractopamine-aminomethyl fluorescein conjugate and mAb against ractopamine. The FPIA demonstrated an LOD of 1 ng/mL, range of detectable concentrations 2.3–50 ng/mL, and high specificity. It can be effectively used to test meat products. Dong et al. [117] developed FPIA for the detection of RAC in pork by synthesizing 10 fluorescein-labeled ractopamine derivatives (tracers) and paring with two pAbs against ractopamine. After careful selection and comparison, a highly sensitive and specific FPIA was developed with an LOD of 0.56 μg/kg for ractopamine in pork.

Zuo et al. [118] developed a method to simultaneously detect clenbuterol (CLB), ractopamine (RAC) and salbutamol (SAL) by hapten microarray-based indirect competitive immunoassay. The BSA conjugates of CLB, RAC and SAL were immobilized on slides precoated with agarose. The corresponding mAbs against these β-agonists, and the standards or samples were introduced for indirect competitive immunoassay. The antigen–antibody binding on the slide was detected using Cy3 dye-labeled secondary antibody. The LODs of CLB, RAC and SAL were 0.09, 0.50 and 0.01 μg/L, respectively. The hapten microarray system can perform high throughput and parallel analysis with high sensitivity and selectivity.

Kong et al. [119] established an integrated microfluidic immunoassay system for high throughput detection of clenbuterol. The 3-layer microchip had eight analysis units in which sample injection, washing, immunoreaction and enzyme catalysis were performed automatically. The competitive immunoassay for clenbuterol was performed. The free clenbuterol in the sample competed with HRP-clenbuterol to bind with the anti-clenbuterol antibody coated on analysis channel. After adding the enzyme substrate, the fluorescence signal was detected by a linear confocal laser-induced fluorescence scanner. The linear range and LOD of clenbuterol were 0–5.0 ng/mL and 0.088 ng/mL, respectively.

## 4. Conclusions and Perspectives

In the past few decades, the illegal use of β_2_-agonists in livestock has received continuous attention worldwide due to its potential threat to public health. In this review, we summarized antibody-based techniques to extract β_2_-agonists from samples, and focused on the development and application of immunological methods in detecting β_2_-agonists (Table 2). Most of the methods can measure β_2_-agonists with high specificity and sensitivity. RIA is replaced largely by nonisotopic immunoassays, due to the difficulties associated with the handling and storage of radioactive reagents and disposal of radioactive waste. ELISA can be used for high-throughput detection of β_2_-agonists, but the sensitivity is moderate and the assay requires a relatively long time. Introduction of nanomaterials in ELISA as enzyme carriers, enzyme mimics and signal reporters may improve the sensitivity, stability and measurement time of the assay. Combination of ELISA with other methods, such as SERS, can also improve the sensitivity of detection. LFIA is fast, easy to perform, not dependent on special equipment and can be carried out on-site to qualitatively or semiquantitatively detect β_2_-agonists. The use of fluorescent nanomaterials, quantum dots and the upconverting phosphors as probes in LFIA can improve the sensitivity of detection. SERS-based LFIA further increase the sensitivity for detecting β_2_-agonists, but instrumentation is required. Chemiluminescence immunoassay (CLIA) and immunosensors, such as SPR-, SERS-, ECL- and electrochemistry-based immunosensors, can detect β_2_-agonists in a short time with high sensitivity, but SPR- and SERS-based immunoassays require special instruments. Current CLIAs for detecting the β_2_-agonist use enzymes to label the secondary Ab and luminescent chemical as substrate to generate light emission. The introduction of luminescent nanoparticles as hemiluminescence probe, such as Au nanoparticles, QDs and magnetic materials, may enhance the sensitivity of the assay. The conventional SPR immunosensors for detecting β_2_-agonists are simple and rapid, but the sensitivity is not adequate. The introduction of Au nanomaterials as labels for Ab and as the amplification tags immobilized on the sensor surface enhances the SPR signal. Continued research should be pursued to explore novel nanomaterials to improve the performance, to generate the SPR chips for recyclable use and exploit miniaturized SPR devices to reduce cost. SERS immunosensors based on nanoprobes detect β_2_-agonists with high sensitivity. However, their stability and reproducibility need to be further improved. Current ECL immunosensors for β_2_-agonist detection mainly use QDs as probes. Synthesis of new ECL probes, development of new biointerface construction and new signal amplification strategies will improve the performance of sensors. In electrochemical biosensors to detect β_2_-agonists, nanomaterials, such as gold nanoparticles and graphene, have been used to modify the electrodes or label antibodies to increase conductivity, electron transfer and signal generation. The performance of the immunosensors may be improved by the construction of labels with a high loading of signal species, introduction of interfacial reaction initiated by functionalized nanomaterials and building a synergistic connection between labels and substrate. More research is needed to develop stable, selective, sensitive, rapid and portable devices for detecting β_2_-agonists.

## Figures and Tables

**Table 1:** Advantages and disadvantages of antibody-based methods for extracting β_2_-agonists from samples.

Methods	Advantages	Disadvantages
Immunoaffinity chromatography	Simple, fast; high separation efficiency and reproducibility	Proteases from animal sample may digest protein A or antibody on the immunoaffinity column. The high-affinity binding of antibodies and antigens may complicate the elution of the antigen.
Immunofiltration	Simple, fast, low cost	Filters are easily clogged by large particles in samples. Contaminating proteins that adhered to the filter will elute with the antigens.
Immunomagnetic separation	Simple, fast, easy to perform; high separation efficiency and reproducibility	Commercial immunomagnetic particles for β_2_-agonists extraction are not available.

**Table 2:** Immunoassays for the detection of β_2_-agonists.

Analytical Technologies	Samples	Analyte	Limit of Detection	References
1. Radioimmunoassay	human plasma	salbutamol	0.5 ng/mL	[30]
	plasma, urine	fenoterol	10–20 pg/mL	[31]
	horse urine	albuterol	28.8 fmol/tube	[32]
	cattle plasma, urine, feces	clenbuterol	7.8 pg/tube	[33]
	bovine liver	clenbuterol, mabuterol, etc.	0.1 μg/kg	[34]
		brombuterol, cimbuterol, etc.	0.3 μg/kg	
2. ELISA				
2.1. Direct competitive ELISA	pork	clenbuterol	0.09 ng/g	[35]
	milk	clenbuterol	0.045 ng/mL	[36]
	feed, milk, swine urine,	clenbuterol	0.03 ng/mL	[39]
	swine serum	salbutamol	0.25 ng/mL	[37]
	swine urine	phenylethanolamine A	0.5 μg/L	[38]
2.2 Indirect competitive ELISA	swine urine, pork	phenylethanolamine A	urine: 0.13 ng/mL, pork: 0.39 ng/g	[40]
	sheep urine	ractopamine	0.35 ng/mL	[41]
	swine and bovine urine	zilpaterol	IC_50_ 3.94 ± 0.48 ng/mL	[42]
	clenbuterol solution	clenbuterol	0.50 ng/mL	[43]
	clenbuterol solution	clenbuterol	0.3 pg/mL	[44]
	salbutamol solution, urine	salbutamol	0.04 ng/mL	[45]
	livestock wastewater	salbutamol	0.66 ng/L	[46]
3. Chemiluminescence immunoassay	pork, liver	salbutamol	0.15 ng/mL	[47]
	swine meat, feed	brombuterol	0.33 pg/mL	[48]
	swine urine	ractopamine	0.97 ng/mL	[49]
4. Lateral flow immunoassay (LFIA)				
4.1. Colorimetric LFIA				
4.1.1. Gold nanoparticle as tracer	swine urine	clenbuterol, ractopamine	0.1 ± 0.01 ng/mL	[50]
	pork muscle	clenbuterol	0.10 ng/g	[51]
	swine urine	salbutamol	1.0 ng/mL	[52]
	swine and turkey meat, cow milk	salbutamol	meat: 3.0 ng/g; milk: 4.0 ng/g	[53]
	swine urine, pork	phenylethanolamine A	5 ng/mL(g)	[40]
	urine, pork	phenylethanolamine A	0.1 ng/mL	[54]
	swine urine	ractopamine	0.13 ng/mL	[55]
	swine urine	ractopamine	2 ng/mL	[56]
	turkey meat, beef liver	ractopamine	0.5 ng/mL	[57]
	swine feed	ractopamine	0.1 ng/g	[58]
	swine urine and feed, milk	clenbuterol	urine: 0.1 ng/mL; feed: 0.2 ng/g; milk: 0.5 ng/mL	[59]
	clenbuterol solution	clenbuterol	2 ppb	[60]
	pork	clenbuterol	0.04 ng/mL	[61]
4.1.2. Other nanoparticles as tracers	swine urine, pork	clenbuterol	urine: 6 ng/mL; pork: 5 ng/mL	[62]
	clenbuterol, ractopamine solution	clenbuterol, ractopamine	clenbuterol: 3 ng/mL; ractopamine: 2 ng/mL	[63]
	swine urine	clenbuterol	3 ng/mL	[64]
	swine urine	ractopamine, salbutamol	ractopamine: 1.0 ng/mL; salbutamol: 3.0 ng/mL	[65]
	pork, swine kidney and bacon	clenbuterol	pork: 3 ng/g; kidney and bacon: 5 ng/g	[66]
	swine feed, pork	ractopamine	feed: 2.0 ng/mL; pork: 1.0 ng/mL	[67]
4.2. Luminescent LFIA	swine urine	clenbuterol	0.037 ng/mL	[68]
	swine urine	clenbuterol	0.22 ng/mL	[29]
	swine urine, feed, pork	clenbuterol, ractopamine, salbuterol	clenbuterol: 0.10 ng/mL; ractopamine: 0.10 ng/mL; salbuterol: 0.09 ng/mL	[69]
	swine urine	ractopamine	7.2 pg/mL	[71]
	swine urine, muscle	ractopamine	0.16 ng/mL	[72]
	pork tissue, urine, feed	clenbuterol	0.01 ng/mL	[73]
	swine urine	ractopamine, clenbuterol	ractopamine: 0.17 ng/mL; clenbuterol: 0.067 ng/mL	[74]
4.3. Other types of LFIA	milk, swine liver, tenderloin	clenbuterol	2 ng/mL	[75]
	swine urine	phenylethanolamine A	0.32 pg/mL	[76]
	swine urine	clenbuterol	0.24 pg/mL	[77]
	swine meat, urine	brombuterol	0.5 pg/mL	[78]
5. Immunosensors				
5.1. Surface plasmon resonance sensors	salbutamol solution	salbutamol	5 ng/mL	[80]
	ractopamine and salbutamol solution	ractopamine, salbutamol	ractopamine: 10 pg/mL, salbutamol: 5 pg/mL	[81]
	swine urine	ractopamine	0.09 ng/mL	[82]
	clenbuterol solution	clenbuterol	0.05 pg/mL	[83]
	bovine urine	clenbuterol	100 fg/mL	[84]
5.2. SERS-based immunosensor	swine urine	clenbuterol	0.1 pg/mL	[85]
	clenbuterol and ractopamine solution	clenbuterol, ractopamine	1.0 pg/mL	[86]
	swine meat and liver, human urine	salbutamol, brombuterol	salbutamol 2.0 pg/mL; brombuterol 1.0.pg/mL	[87]
	clenbuterol solution	clenbuterol	0.22 fg/mL	[88]
	clenbuterol solution	clenbuterol	0.68 pg/mL	[89]
5.3. Electrochemiluminescence immunosensor	pork and liver	salbutamol	8.4 pg/mL	[91]
	pork and liver	salbutamol	17 pg/mL	[92]
	pork and feed	ractopamine	1.7 pg/mL	[93]
	pork	salbutamol	0.17 pg/mL	[94]
	pork and feed	brombuterol	1.5 pg/mL	[95]
	pork	brombuterol	0.3 pg/mL	[96]
	pork	ractopamine	2.6 pg/mL	[97]
	pork and swine feed extract	brombuterol	0.31 pg/mL	[98]
5.4. Electrochemical immunosensor	porcine serum	salbutamol	0.2 fg/mL	[99]
	serum	salbutamol	7 pg/mL	[102]
	swine urine	ractopamine	2.3 pg/mL	[100]
	clenbuterol solution	clenbuterol	0.12 ng/mL	[101]
	pork	salbutamol, ractopamine, clenbuterol	salbutamol: 1.44 pg/mL, clenbuterol: 1.38 pg/mL, ractopamine: 1.52 pg/mL	[103]
	pork, feed	salbutamol	0.04 ng/mL	[105]
	salbutamol solution	salbutamol	0.06 ng/mL	[106]
	salbutamol solution	salbutamol	0.03 ng/mL	[107]
	bovine hair	clenbuterol	0.008 ng/mL	[108]
	milk	clenbuterol	0.196 ng/mL	[109]
	clenbuterol solution	clenbuterol	0.076 ng/mL	[111]
	swine feed	clenbuterol	0.25 ng/mL	[110]
6. Other types of immunoassays	human urine	salbutamol	28 fg/mL	[113]
	water, urine	salbutamol	0.65 fmol/ML	[114]
	pork	salbutamol, ractopamine	sabutamol: 0.051 μg/kg, ractopamine: 0.02 μg/kg	[115]
	turkey meat	ractopamine	1 ng/mL	[116]
	pork	ractopamine	0.56 μg/kg	[117]
	clenbuterol, ractopamine and salbutamol solution	clenbuterol, ractopamine, salbutamol	clenbuterol: 0.09 μg/L; ractopamine: 0.50 μg/L; salbutamol: 0.01 μg/L	[118]
	swine urine	clenbuterol	0.088 ng/mL	[119]

## Data Availability

Not applicable.
